# Temporoparietal Fascia Flap (TPFF) in Extended Endoscopic Transnasal Skull Base Surgery: Clinical Experience and Systematic Literature Review

**DOI:** 10.3390/jcm13237217

**Published:** 2024-11-27

**Authors:** Martina Offi, Pier Paolo Mattogno, Ginevra Federica D’Onofrio, Simona Serioli, Federico Valeri, Giuseppe Maria Della Pepa, Vincenzo Arena, Claudio Parrilla, Sabrina Chiloiro, Francesco D’Argento, Marco Gessi, Alessandro Pedicelli, Liverana Lauretti, Gaetano Paludetti, Jacopo Galli, Alessandro Olivi, Mario Rigante, Francesco Doglietto

**Affiliations:** 1 Faculty of Medicine and Surgery, Università Cattolica del Sacro Cuore, 00168 Rome, Italy; ginevra.federica.donofrio@gmail.com (G.F.D.); simo195@hotmail.it (S.S.); federicovaleri97@gmail.com (F.V.); vincenzo.arena@policlinicogemelli.it (V.A.); sabrina.chiloiro@policlinicogemelli.it (S.C.); marco.gessi@policlinicogemelli.it (M.G.); alessandro.pedicelli@policlinicogemelli.it (A.P.); liverana.lauretti@unicatt.it (L.L.); gaetano.paludetti@policlinicogemelli.it (G.P.); jacopo.galli@policlinicogemelli.it (J.G.); alessandro.olivi@policlinicogemelli.it (A.O.); 2 Neurosurgery, Fondazione Policlinico Universitario A. Gemelli IRCCS, 00168 Rome, Italy; giuseppemaria.dellapepa@policlinicogemelli.it; 3 Neurosurgery, Sant’Eugenio, 00168 Rome, Italy; 4 Neurosurgery, Spedali Civili Hospital, 25123 Brescia, Italy; 5 Pathology, Fondazione Policlinico Universitario A. Gemelli IRCCS, 00168 Rome, Italy; 6 Otolaryngology, Fondazione Policlinico Universitario A. Gemelli IRCCS, 00168 Rome, Italy; claudio.parrilla@policlinicogemelli.it (C.P.); mario.rigante@policlinicogemelli.it (M.R.); 7 Endocrinology, Fondazione Policlinico Universitario A. Gemelli IRCCS, 00168 Rome, Italy; 8 Neuroradiology, Fondazione Policlinico Universitario A. Gemelli IRCCS, 00168 Rome, Italy; francesco.dargento@policlinicogemelli.it

**Keywords:** cranial base reconstruction, endoscopic transnasal surgery, neurosurgery, skull base reconstruction, temporo-parietal flap, vascularized flap

## Abstract

**Background and Objectives:** The temporoparietal fascia flap (TPFF) has recently emerged as an option for skull base reconstruction in endoscopic transnasal surgery when vascularized nasal flaps are not available. This study provides a systematic literature review of its use in skull base surgery and describes a novel cohort of patients. **Methods:** PRISMA guidelines were used for the review. Patients undergoing skull base reconstruction with TPFF in our center from May 2022 to April 2024 were retrospectively included. Data were collected on pre- and post-operative clinical and radiological features, histology, surgical procedures, and complications. **Results:** Sixteen articles were selected, comprising 42 patients who underwent TPFF reconstruction for treatment of complex skull base pathologies. In total, 5 of 358 patients (0.9%) who underwent tumor resection via endoscopic transanal surgery in the study period in our institution required TPFF. All had been previously treated with surgery and radiation therapy for different pathologies (three chordomas, one giant pituitary neuroendocrine tumor (PitNET), and one sarcoma). Post-operative complications included CSF leak, which resolved after flap revision, and an internal carotid artery pseudoaneurysm requiring endovascular embolization. **Conclusions:** TPFF is an effective option for skull base reconstruction in complex cases and should be part of the armamentarium of the skull base surgeon.

## 1. Introduction

The indications for transnasal endoscopic surgery have expanded considerably during the last two decades, and to date, thanks to the development of the extended endonasal approaches (EEAs), the anterior, middle, and posterior cranial fossae have become accessible through the transnasal route [1,2,3,4,5,6]. These approaches provide direct access to pathologies, while simultaneously minimizing the need for large craniotomies or more invasive surgical techniques [7,8,9,10,11]. As a result, the use of EEAs in skull base surgery has allowed the treatment of more extensive and complex pathologies, along with a corresponding increase in the size of osteo-dural defects created during resection [12,13]. However, these advancements have introduced new challenges in the reconstruction process, making it essential to develop effective strategies to minimize complications and optimize patient recovery. A key focus of these strategies is the prevention of cerebrospinal fluid (CSF) leaks, infections, and long-term complications, particularly in patients who have received radiotherapy [14,15]. Reconstructive techniques have evolved alongside the expansion of EEAs, progressing from the repair of smaller defects, which can be managed with simple bone and fat grafts, to the need for more sophisticated methods for larger bony defects. The choice of reconstruction technique is determined by various factors, including the size of the defect, its anatomical location, and the type of flap available.

In anterior skull base reconstruction, local nasal flaps are generally the preferred option, due to their size, location, and simplicity to be rotated [16,17]. Since its introduction in 2006, the Hadad–Bassagasteguy flap has consistently demonstrated excellent outcomes in terms of preventing CSF leaks and promoting healing [16]. For smaller defects, inferior and middle turbinate flaps can be used. However, their effectiveness is limited to modest approaches, as they may not provide sufficient coverage for larger or more complex defects. Another option is the lateral nasal wall flap, which is ideal for defects in the lateral wall and anterior nasal regions. This flap provides good vascularity, but its application is constrained when addressing larger or more posterior defects [17].

In scenarios where local flaps are not viable, available, or appropriate, alternative reconstruction techniques are needed. Regional vascularized flaps, such as pericranial, palatal, and temporo-parietal fascial flaps (TPFF), offer viable alternatives. These are harvested from anatomically distant but well-vascularized areas, making them suitable for more extensive defects or when local options are exhausted. The pericranial flap is a commonly used regional flap, especially to cover large cranial defects or areas requiring substantial bony reconstruction. The palatal flap, which can be harvested from the hard or soft palate, is another option for defects of the lower skull base defects or nasal floor. However, its use is associated with potential complications related to palatal resection and is generally reserved for cases where other options are not feasible. Additionally, the palatal flap may not be suitable for very large defects, as its size and vascularity may not provide sufficient coverage [17].

The TPFF has become increasingly popular for reconstructing large and complex skull base defects, particularly in cases involving multiple resections, recurrent tumors, or a history of radiotherapy. This flap, derived from the temporo-parietal fascia, is widely recognized for its utility in head and neck reconstruction, including otolaryngology and plastic surgery [18,19]. It offers significant flexibility and durability, making it particularly suitable for challenging defects [20]. However, harvesting the TPFF is technically demanding and is often associated with higher morbidity compared to local flaps. Despite these challenges, it remains an invaluable option when local or other regional flaps are inadequate.

Thus, the selection of a reconstructive flap in skull base surgery depends on various factors, such as the defect’s size and location, the patient’s disease, and the availability of viable, vascularized tissue. Local flaps, particularly the Hadad–Bassagasteguy flap, are typically the first choice, while regional flaps like the TPFF provide alternatives for more extensive or complex defects.

As the literature still reports relatively limited data, we carried out a systematic review of TPFF in skull base reconstruction and report our recent experience with its use in EEAs.

## 2. Materials and Methods

### 2.1. Systematic Review

PubMed and Cochrane databases were systematically searched using specific keywords related to TPFF and skull base reconstruction: (temporo parietal OR temporo-parietal OR temporoparietal OR TPFF) AND flap. The PRISMA algorithm was employed [21]. Two independent reviewers (M.O., S. S.) manually screened abstracts and papers. Data extracted included demographic information, treatment history, indications, histology of primary lesions, intra- and post-operative complications, radiological assessments, progression-free survival (PFS), and outcomes. Surgical techniques for TPFF, including flap preparation and transposition methods, were evaluated. Eligibility criteria included English-language case reports detailing TPFF reconstruction for anterior skull base procedures. Reports involving middle cranial base reconstructions, lateral approaches, or flap transpositions for extracranial reconstruction were excluded.

### 2.2. Case Series

Data on the 5 patients who underwent skull base reconstruction with TPFF in EEAs at Fondazione Policlinico Agostino Gemelli University Hospital—IRCCS (Rome, Italy) from May 2022 to April 2024 was retrospectively collected.

Each patient provided informed consent, and the procedures were carried out in accordance with the standards of the local ethics committee and the Helsinki Declaration of 1975, revised in 1983. Data on demographics, tumor type, surgical procedure performed, complications, early postoperative course, and follow-up were collected.

### 2.3. Surgical Technique

**Figure 1:**
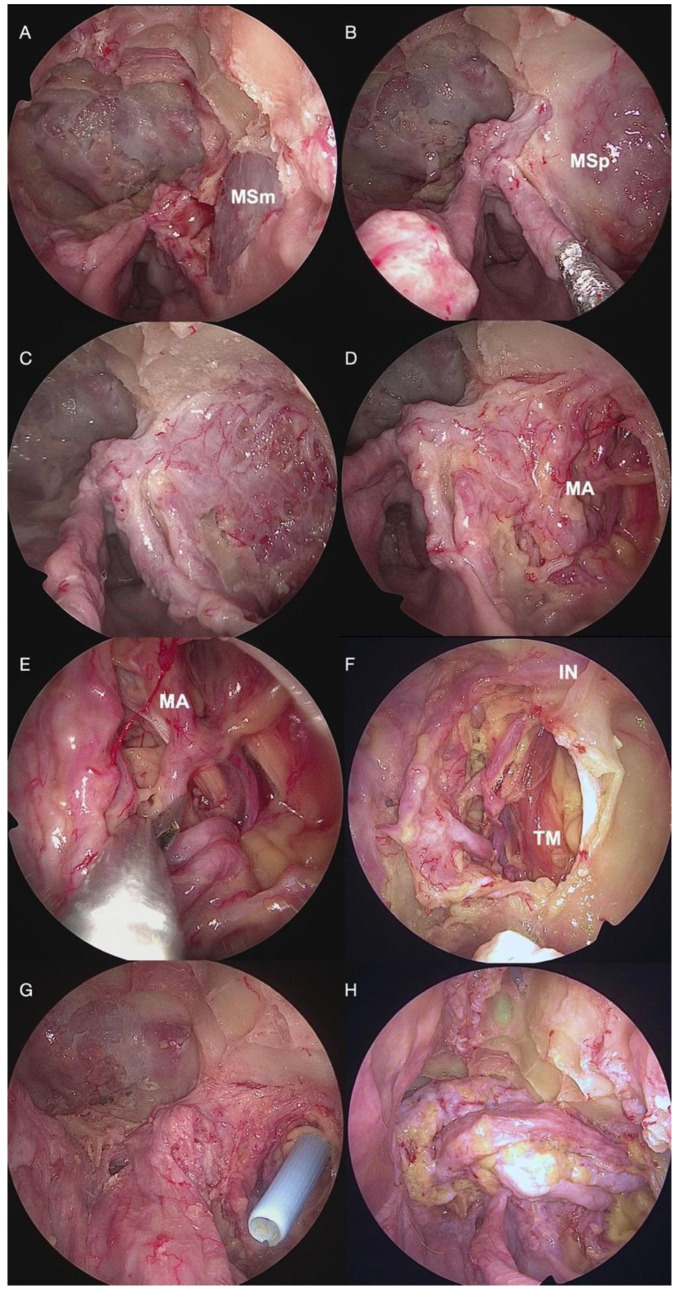
**First and final nasal phases.** (**A**) Opening of the maxillary sinus. (**B**) Opening of the anterior wall of the maxillary sinus. (**C**) Opening of the posterior wall of the maxillary sinus. (**D**) Identification of the maxillary artery. (**E**) Sectioning of the maxillary artery. (**F**) Identification of the temporal muscle, the infraorbital nerve and artery. (**G**) Passage of the Blue Rhino^®^ guide (*see text for details*) for flap transposition. (**H**) Positioning of the folded TPFF to cover the bone defect. *Abbreviations:* IN, infraorbital nerve; MA, maxillary artery; MSm, medial wall of maxillary sinus; MSp, posterior wall of maxillary sinus; TM, temporal muscle.

**Figure 2:**
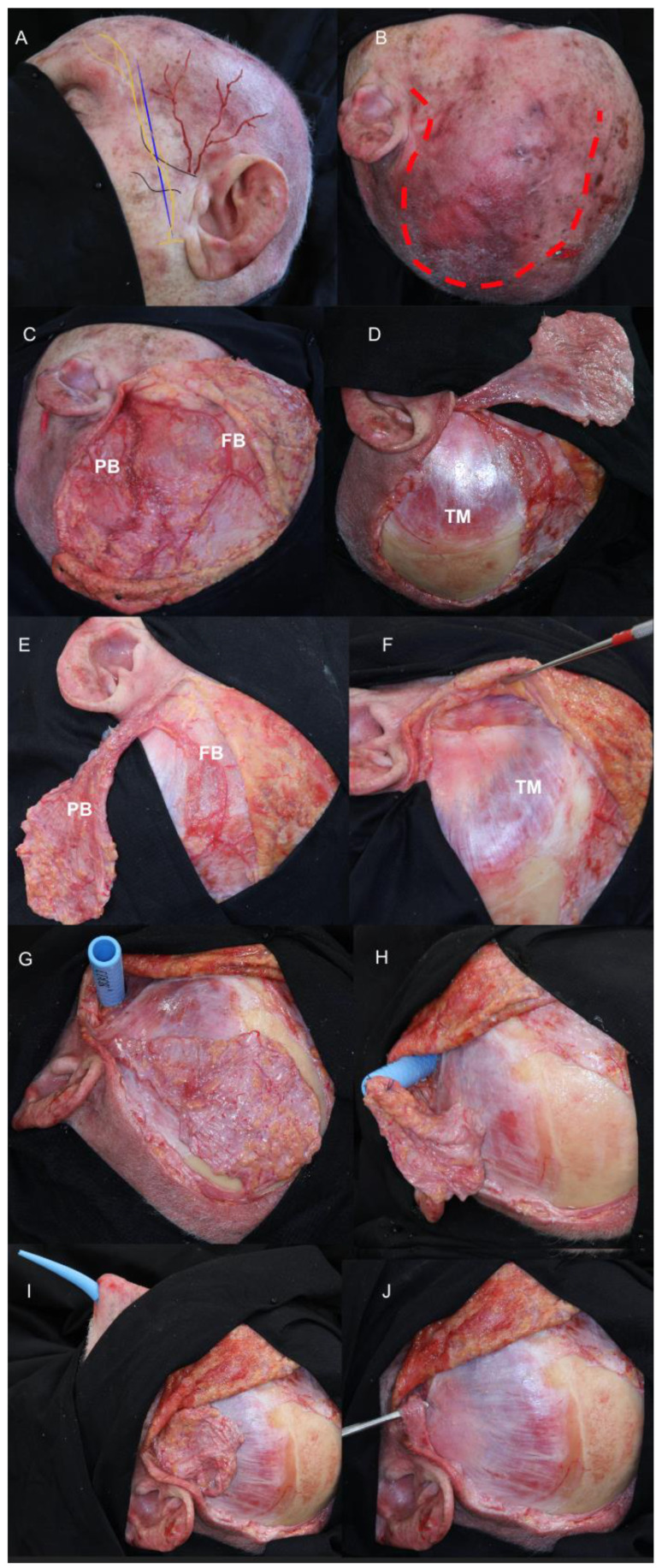
**Cranial phases.** (**A**) The lines depict the different landmarks on the skin. Blue: Pitanguy’s line, Yellow: Temporal branch of the facial nerve, Black: Zygomatic arch, Red: STA. (**B**) Red dashed line: Incision. (**C**) After subcutaneous dissection, identification of STA, frontal (FB) and posterior branches (PB). (**D**) Incision of the flap utilizing the posterior branch of the STA with dissection from the temporal muscle fascia (TM). (**E**) TPFF flap with identification of FB and PB branches of the STA. (**F**) Subfascial dissection. (**G**) Placement of the Rhino percutaneous tracheostomy dilator in the infratemporal fossa. (**H**) Suturing of the TPFF to the guide. (**I**) Progressive transposition of the TPFF through the infratemporal fossa, pterygoid fossa, and into the nasal cavity by pulling the guide from the maxillary sinus into the nasal cavity. (**J**) Complete transposition of the TPFF without compression or bending of the flap base. *Abbreviations:* FB, frontal branch of superficial temporal artery; FC, facial nerve; PB, parietal branch of superficial temporal artery; STA, superficial temporal artery; ZA, zygomatic arch; TM, temporal muscle.

The surgical technique was detailed in a step-by-step dissection of an anatomical specimen, with the arterial system injected with red-dye silicon, in the SURGEM laboratory (Human Anatomy and Forensic Medicine Laboratory at Fondazione Policlinico Universitario A. Gemelli IRCCS) (Figure 1 and Figure 2) and reported herein.

In the first endoscopic nasal phase, after middle turbinectomy and ethmoidectomy, an ipsilateral maxillary antrostomy was performed (Figure 1A,B), the posterior wall of the maxillary sinus was opened (Figure 2C), and the infratemporal fossa (ITF) was exposed. The infraorbital nerve, maxillary artery and its branches, and temporal muscle were identified (Figure 2D–F).

In the cranial phase, the localization of the superficial temporal artery (STA) with its two branches and the temporal branch of the facial nerve are drawn on the skin (Figure 2A). A fronto-temporo-parietal curvilinear incision is performed to include the planned TPFF and allow for an interfascial dissection (Figure 2B). The skin and subcutaneous tissue are dissected, identifying the superficial temporal artery, with its frontal and posterior branches (Figure 2C). The flap is harvested along the largest posterior branch of the STA, sacrificing the frontal and minor ones.

Utilizing a periosteal elevator, the incised portion, along with the attached periosteum and overlying subgaleal layer, is stripped as a single-thickness sheet (Figure 2D,E). Proximally, an interfascial dissection of the temporalis muscle is performed, and the zygomatic arch is exposed (Figure 2F). Subsequently, the deep temporal fascia is opened, and a tunnel is created below the zygomatic arch to gain access to the ITF under endoscopic visualization (Figure 1G). The tunnel is gently dilated using a percutaneous tracheostomy dilator (Ciaglia Blue Rhino^®^ Advanced Percutaneous Tracheostomy kit, Cook Medical) (Figure 2G–J). A large bore flexible tube is subsequently passed over the dilators, with the distal end of the flap sutured to the guide wire (Figure 2H). The flap is passed through this tunneled tube into the maxillary sinus and then the nasal cavity, ensuring that the pedicle’s integrity is maintained and avoiding twisting (Figure 1G,H and Figure 2G–J).

In the final nasal phase, the flap is positioned over the skull base defect, taking care that it is well positioned over the surrounding bone. If necessary, it can be sutured (e.g., to the rhinopharynx mucosa). Nasal packing is used as appropriate. The cranial incision is reapproximated with Donati sutures, and a subgaleal drain is kept in situ for 48 h.

## 3. Results

### 3.1. Systematic Review

The initial search yielded 564 publications. Of these, 448 were excluded according to the PICOS criteria, as outlined by the PRISMA guidelines: (P) 138 were excluded because they were based on cadaveric specimens or lacked Patient data; (I) 267 were excluded for focusing on middle cranial base reconstructions, lateral approaches, or flap transpositions for extracranial reconstruction, which were outside the scope of the Intervention under review; (O) 43 were excluded as they did not report patient Outcomes, making it impossible to assess the effectiveness of the intervention; (C, S) The lack of a Comparison or control group, as well as issues related to Study design, were not exclusion criteria for this review. This exclusion process helped refine the studies included in our review to ensure relevance and quality of evidence.

**Figure 3:**
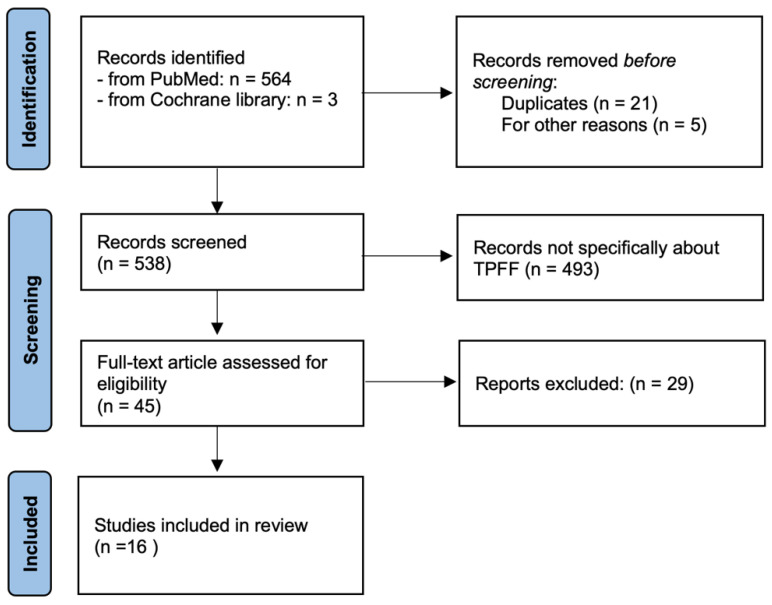
PRISMA flowchart of the systematic literature review.

The final selection included 16 studies describing 42 patients who underwent skull base reconstruction using TPFF (Figure 3).

Data from the included articles are summarized in Table 1. The mean age of patients was 48 years (range: 2–77). Most (60%, 25/42) had previously received radiotherapy.

The indications for using the TPFF varied. Disease recurrence was the most common indication, accounting for 14 patients (33%). An extended surgical approach was required in nine patients (21%), while seven patients (17%) needed the flap due to recurrent CSF leaks. Radionecrosis or necrosis necessitated the procedure in four patients (9.5%), and other reasons, such as exposure of the internal carotid artery (ICA) or unavailability of local nasal flaps, accounted for eight patients (19%).

Technically, most patients (35/42, 83%) were treated with the TPFF variant, while the remaining 16.7% (7 patients) received the temporoparietal temporalis muscle flap (TPTMMF). The method of flap transposition also varied, with 54.8% of patients (23 of 42) undergoing endonasal transposition and 45.2% (19 of 42) undergoing transposition via craniotomy.

Complications were experienced for 8 of 38 (19.5%) patients. These included cerebrospinal fluid leak (2 patients, 4.8%), hematoma (3 patients, 7.1%), intracranial infection (2 patients, 4.8%), and injury to the superficial temporal artery (1 patient, 2.4%). Flap necrosis was not reported as a complication. Lumbar drainage was used in 9.5% of cases (4 of 42).

Follow-up (FU) data were available for 39 patients, with a mean period of 21 months. Disease status was specified in 35 patients: 25 (71%) showed no evidence of disease, 2 (6%) were alive with disease, and 8 (23%) had died from disease.

### 3.2. Case Series

Of 358 patients who underwent tumor resection via endoscopic transanal surgery at the Neurosurgery and Otolaryngology Units of Fondazione Policlinico Agostino Gemelli University Hospital—IRCCS (Rome, Italy) from May 2022 to April 2024, five (0.9%) required a TPFF (Table 2).

The cohort comprises three males and two females, with a median age of 55 years (range 47–78). Pathology documented clivus chordoma in two patients, one non-functioning giant pituitary neuroendocrine tumor (PitNET), one silent ACTH PitNET, and a high-grade spindle cell mesenchymal tumor. Three patients had undergone adjuvant radiotherapy and chemotherapy prior to surgery requiring TPFF. The TPFF was used in all five cases due to the unavailability of nasal flaps. All patients underwent endoscopic extended transnasal approaches. In all patients, the integrity of the vascular pedicle of TPFF was preventively studied with brain angioMRI or angioCT.

Indications included disease recurrence (with the need for a combined approach, with the use of pericranial flap intracranially and TPFF nasally—Patient 1); CSF leak (Patients 4–5); hydrocephalus and meningitis with no evidence of leak, but a suboptimal previous nasal flap (Patient 3); ICA pseudoaneurysm, which bled despite first TPFF positioning and was actually the cause of its necrosis—Patient 2). Two patients also had skull base osteomyelitis, most probably radio-induced (Patients 2 and 5): both had revision surgery to optimize TPFF adhesion to the skull base due to evidence of partial dehiscence.

In two patients (2 and 3), the TPFF was harvested bilaterally: in Patient 3, before the study period, to address a CSF leak; in Patient 2, due to necrosis of the previously positioned flap.

**Figure 4:**
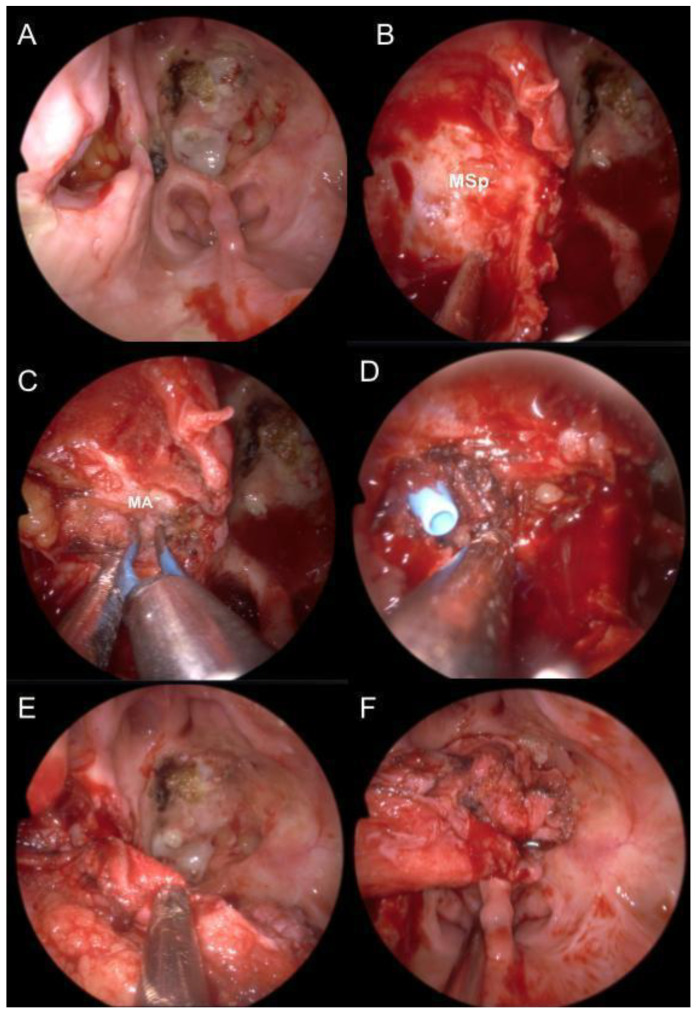
**First and final nasal phases.** (**A**) Defect of the previous surgery, with necrosis of the previous Hadad septal nose flap and recurrent CSF fistula. (**B**) Opening of the posterior wall of the maxillary sinus. (**C**) Identification and coagulation of the maxillary artery. (**D**) Passage of the Blue Rhino^®^ guide for flap transposition. (**E**) Preparation of the folded TPFF. (**F**) Positioning of the folded TPFF to cover the bone defect. *Abbreviations:* MA, maxillary artery; MSp, posterior wall of maxillary sinus.

**Figure 5:**
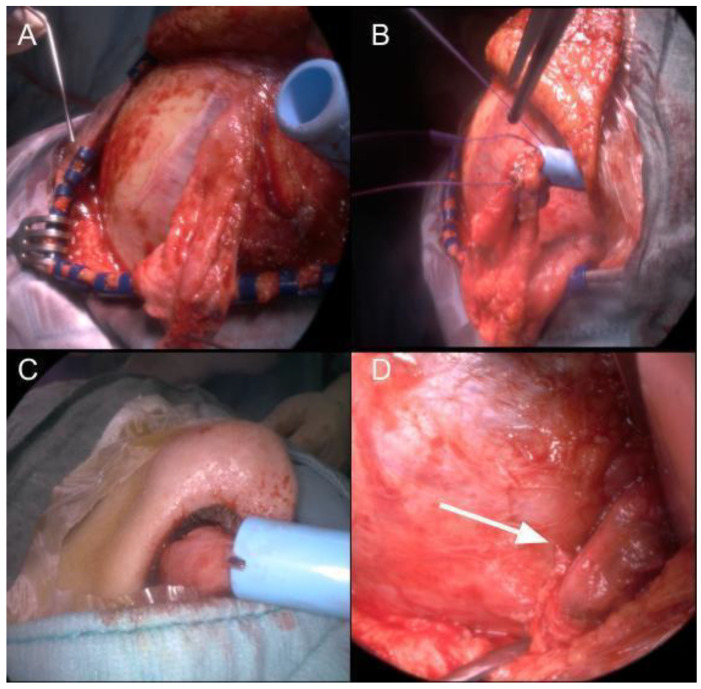
**Cranial phases.** (**A**) Preparation and incision of the flap utilizing the posterior branch of the STA with dissection from the temporal muscle fascia (TM) and placement of the Rhino percutaneous tracheostomy dilator in the infratemporal fossa. (**B**) Suturing of the TPFF to the guide. (**C**) Progressive transposition of the TPFF into the nasal cavity by pulling the guide until the guide completely comes out of the nasal cavity, then the suture is cut. (**D**) Complete transposition of the TPFF without compression or bending of the flap base (*white arrow*). *Abbreviations:* TM, temporal muscle.

In all patients, the TPFF was transposed via an infratemporal fossa (ITF) route. The procedure involved an initial endoscopic nasal phase with middle turbinectomy, ethmoidectomy, and ipsilateral maxillary antrostomy to expose the ITF. In the cranial phase, a fronto-temporo-parietal incision was made to harvest the TPFF, which was then successfully transposed through the ITF in each case. The technique used is shown in detail in Figure 4 and Figure 5.

No intraoperative complications were reported and no ruptures of superficial temporal arteries occurred, as documented in other case series [36]. Due to the addition of the cranial phase for TPFF harvesting, the surgical time was approximately 2 h longer than the time typically required for the endoscopic phase alone. In the operating room, the simultaneous work of two teams was necessary: one for the cranial phase and one for the endoscopic phase.

All patients had evidence of local alopecia, along with and aside from the skin incision, but there were no signs of skin necrosis or defects in the healing of the surgical wound. All patients reported discomfort in chewing for the first days and sometimes weeks after surgery. None of the five patients required placement of a lumbar drain.

The mean follow-up is 12 months (range 7–17 months). Two patients died due to disease progression despite maximal therapy, one patient died due to unrelated cardiovascular disease, and two are alive with disease.

## 4. Discussion

The expanded applications of endoscopic endonasal approaches for the management of complex skull base lesions have brought about significant challenges in reconstruction. The preferred options involve the multilayer technique and the use of a vascularized pedicled flap, as these were shown to produce superior results in terms of reducing the rate of complications and promoting rapid healing of the surgical area. This is particularly important in case of large defects or for patients who received multiple procedures, preoperative radiotherapy, chemoradiotherapy or postoperative radiotherapy [36,37,38,39].

The vascularized nasal septal flap described by Hadad et al. [16] remains the first option for reconstruction, as it leads to a significant decrease in CSF leak, is relatively simple to harvest in the surgical corridor, and is characterized by minimal additional morbidity. There are situations, however, in which this option is not available such as previous surgery with no preservation of the vascular pedicle or tumor infiltration. The TPFF represents an excellent rescue strategy in case of extensive surgical approaches with large defects, revision surgery for recurrent disease, or to deal with complications of prior treatments.

This comprehensive analysis of TPFF use in skull base reconstruction provides valuable insights into its versatility and efficacy in addressing various clinical challenges. The heterogeneity of patient demographics, lesion characteristics, and surgical indications underscores its applicability across diverse clinical scenarios.

The TPFF has proven to be a reliable option to achieve long-lasting skull base reconstruction, particularly for clival and parasellar defects, its robust and elongated vascular pedicle, and thanks to its considerable surface area, thinness, and pliability, it is easily adaptable to different and irregular surfaces.

In 2007, Fortes et al. [20] described the so-called “transpterygoid” transposition of TPFF to allow its use in endoscopic skull base surgery: the flap is moved from the temporal to the infratemporal fossa, advancing it into the nasal cavity through the transpterygoid tunnel. These authors described patients who had undergone radiotherapy and with large postoperative defects, noting the resolution of CSF leaks with rapid mucosalization of the flaps without postoperative complications [20].

This innovative approach has since been adopted and elaborated by several authors, including Bolzoni Villaret et al. [24], Safavi-Abbasi et al. [26], Fernandez-Miranda et al. [28], and Arosio et al. [30].

In our systematic review of 16 studies involving 42 patients, we found that the TPFF was employed in a variety of clinical contexts, with disease recurrence (33%), extended surgical approaches (21%), and recurrent CSF leaks (17%) being the most common indications for its use.

In the last few years, some authors have proposed modifications to the flap: the temporoparietal temporalis myo-fascial flap (TPTMFF) integrates deeper fascial and pericranial layers within the pedicle, along with a strip of temporalis muscle from the contralateral side at the tip of the flap. This technique, described by Kawasar et al. [31], Land et al. [33], and Kimura et al. [35], involves a bi-coronal incision.

Some additional uses of TPFF have been reported. Iwami et al. [29] described using the TPFF to wrap the petrous portion of the ICA in two cases after combined tumor resection and in a case of skull base radiation necrosis after proton beam therapy. Similarly, a TPFF was used in two cases of radio-induced skull base osteomyelitis in our series, in one case associated with a paraclival ICA pseudoaneurysm revascularization due to dislodgement of the coils that had been positioned after the first surgery at another center.

Collar et al. [23] also described using TPFF for auricular lip reconstruction, re-contouring temporal-zygomatic deformities secondary to temporalis transposition, addressing cutaneous and mucosal oncologic defects, and for orbital and skull base reconstruction.

Other alternative methods of flap transposition that have been explored and generally used for anterior skull base defects reconstruction if the pericranium flap is not available, were through craniotomies, as shown by Hasegawa et al. [22], Collar et al. [23], Ferrari et al. [40], Tabano et al. [27], Iwami et al. [29], Idriceanu et al. [34], and Bresson et al. [41]. These approaches typically entail a nearly complete bicoronal incision to maximize the length of the TPFF. A small frontal craniotomy may then be performed to expose the anterior fossa floor, through which the TPFF is introduced into the nasal cavity via the craniotomy and extradural corridor.

Our experience at Fondazione Policlinico Agostino Gemelli University Hospital aligns with the findings in the literature. Over a 2-year period, TPFF was utilized in only 5 of 358 patients who underwent tumor resection via endoscopic transnasal surgery. These cases included recurrent clival chordomas, non-functioning giant PitNET, and high-grade spindle cell mesenchymal tumors, following prior adjuvant therapies, such as radiation and chemotherapy, in three of the five patients. TPFF was indicated primarily due to the unavailability of nasal flaps, and in all cases, it was successfully harvested via an infratemporal fossa approach. We opted not to propose a transposition via craniotomy because this approach is more invasive and significantly increases surgical time, even though this aspect has not yet been specifically analyzed in clinical studies. While the craniotomy route has often been chosen in many cases to reduce the risk of TPFF pedicle compression [41], we have not observed any instances of pedicle compression using the transnasal transposition approach. Although the most recent publication on craniotomy-based transposition reported no long-term complications, we believe that the trans-infratemporal fossa corridor remains a safer and more efficient option, minimizing both invasiveness and surgical time. Furthermore, the cranial phase of flap harvesting adds approximately two hours to the overall surgical time, which can be a significant consideration in certain clinical settings, particularly when the goal is to minimize the duration of surgery and patient recovery time.

No intraoperative complications were reported, and the flap functioned well in all patients, although two patients did experience partial flap dehiscence during follow-up due to the compromised tissue from prior radiation therapy and required surgical revision. One notable modification in our practice was the use of bilateral TPFF harvest in two patients (Cases 2 and 3) to address large defects or complications, including flap necrosis (Case 2) and a suboptimal prior flap (Case 3). This emphasizes the versatility of TPFF and the potential to adapt the technique to meet the specific needs of each patient.

In conclusion, a TPFF was selected in approximately 0.9% of patients approached via the endoscopic endonasal route over a two-year period at our Institution. This highlights the selective but crucial use of TPFF to manage complex cases, particularly in the setting of previous radiotherapy and tumor recurrence, and should be considered as part of a skull base surgeon’s armamentarium.

### Limits of the Study

The retrospective nature of the present investigation may have introduced the inherent biases and limitations associated with retrospective data analysis, potentially impacting the robustness of the findings. Furthermore, the relatively small sample size of the study population may restrict the generalizability of the results but is justified by the highly selected cases in which such a flap can be used. Additionally, potential biases, such as selection bias stemming from the inclusion criteria or confounding variables that were not fully accounted for, should be considered and discussed to provide a comprehensive understanding of the scope and implications of the study.

Nonetheless, the systematic review compensates for the biases of a small but relatively significant case series.

## 5. Conclusions

The systematic review and our series support the efficacy and versatility of TPFF for the reconstruction of the skull base, even in challenging situations. TPFF is of paramount importance when local nasal options are not available, as in patients who have previously undergone multiple treatments, frequently including radiation and chemotherapy. It has the advantage of providing vascularized tissue at the skull base, which can act as a barrier against CSF leak but also deal with radio-induced inflammatory changes.

The surgical technique is peculiar and includes recent variations that have been described to deal with different scenarios. It is a technique that should be part of the armamentarium of every skull base surgeon.

## Figures and Tables

**Table 1:** Overview of studies detailing patient demographics, lesion histology, surgical indications, types of flaps used, complications, and follow-up.

Author, Year of Publication	N	Flap Type	Transposition	Histology	Indication	Complications
Hasegawa, 1995 [22]	10	TPTMMF	Craniot	M, SCC, AC, RS	Large Skull base defect	CSF leak, subdural hematoma
Fortes, 2007 [20]	2	TPFF	Nasal	CC	CSF, RTN	None
Collar, 2012 [23]	1	TPFF	Craniot	Neuroblastoma	CSF	NA
Bolzoni Villaret, 2013 [24]	5	TPFF	Nasal	CC, nasopharyngeal carcinoma, AC	Recur	None
Rastatter, 2016 [25]	1	TPFF	Combined	CC	CSF	NA
Safavi-Abbasi, 2016 [26]	1	TPFF	Combined	Malignant PNST	Large Skull base defect	None
Tabano, 2019 [27]	1	TPFF	Craniot	SCC	Recur	None
Fernandez-Miranda, 2020 [28]	1	TPFF	Nasal	Cph	CSF	None
Iwami, 2020 [29]	3	TPFF	Combined	CC, angioma, AC	Recur, ICA, RN	ICA pseudoaneurysm, epidural abscess
Arosio, 2021 [30]	1	TPFF	Nasal	AC	RN	None
Kawsar, 2021 [31]	6	TPTMMF	Nasal	ACTH-PitNET, CC, Cph, AC, OGM	CSF	None
London, 2021 [32]	1	TPTFF	Nasal	AC	Recur	NA
Land, 2022 [33]	2	TPTMMF	Nasal	ACTH-PitNET, CC	CSF	None
Idriceanu, 2023 [34]	1	TPTFF	Nasal	C	RN	NA
Kimura, 2023 [35]	1	TPTFF	Nasal	OGM	Recur	NA
Bresson, 2024 [36]	5	TPTFF	Combined	S, C, ITAC, nITAC	Large Skull base defect	injured STA, intraconal hematoma, CSF leak, subdural empyema

*Abbreviations:* AC, adenoid cystic carcinoma; ACTH-PitNET (ACTH-Pituitary neuroendocrine tumor), ACTH pituitary adenoma; C, chondrosarcoma; CC, Clival chordoma; Cph, Craniopharyngioma; CSF, Cerebrospinal Fluid leak; CT, Computed Tomography; ICA, Internal Carotid Artery; ITAC, Intestinal-Type Adenocarcinoma; M, meningioma; MRI, Magnetic Resonance Imaging; nITAC, Non-Intestinal-Type Adenocarcinoma; OGM, olfactory groove meningioma; PNST, peripheral nerve sheath tumor; RS, rhabdomyosarcoma; RT, Radiotherapy; S, sarcoma; SCC, squamous cell carcinoma; STA, Superficial Temporal Artery; TPFF, Temporoparietal Fascial Flap; TPTMMF, Temporoparietal Temporalis Muscle Flap.

**Table 2:** Overview of the case series detailing patient demographics, histology, previous treatments, surgical indications, TPFF side and transposition technique, complications, follow-up, and outcome.

Pt	Age/ Sex	Histology	No. Surgeries Pre-TPFF	Previous RT	Previous CT	Indications	TPFF Side	Transposition	Complications	FU (m)	Outcome
1	49/F	S	2	No	No	Recur	R	Combined	None	17	DOD
2	78/M	CC	1 and 2	Yes (HT)	Yes	SBO, ICA pseudo-an	R, L	Nasal	TPFF necrosis	16	AWD
3	49/F	CC	7	Yes (HT)	Yes	Mening, H	(L*)R	Nasal	None	10	DOD
4	48/M	Giant NF PitNET	6	No	No	CSF	L	Nasal	CSF, H, TPFF dehis	10	AWD
5	51/M	ACTH-PitNET	5	Yes (FSRT, 2 g-knife, HT)	Yes	SBO, CSF	L	Nasal	TPFF dehis	7	D: CV

*Abbreviations:* Ad, A1, anterior cerebral artery; an, aneurysm; ACTH-PitNET (ACTH-Pituitary neuroendocrine tumor), ACTH pituitary adenoma; AWD, alive with disease; CC, Clival chordoma; CSF, Cerebrospinal Fluid leak; CT, chemotherapy; CV, Cardiovascular disease; D, died of; dehis, dehiscence; DOD, died of Disease; g-knife, gamma-knife radiosurgery; H, Hydrocephalus; HT, hadron therapy; FSRT, Fractionated Stereotactic Radiotherapy; FU, follow-up; Hypopit, hypopituitarism; ICA, Internal Carotid Artery; m, months; Mening, meningitis; N., number of; RT, Radiation Therapy; S, sarcoma; SBO, Skull base osteomyelitis; TPFF, Temporoparietal Fascial Flap. *A left TPFF was previously used in the patient by FD, outside the study period, in another institution.
